# *MIF* Promoter Variant rs755622 (−173G/C) in Younger and Older Turkish Adults: An Exploratory Cross-Sectional Genetic and in Silico Analysis

**DOI:** 10.3390/ijms27177818

**Published:** 2026-08-31

**Authors:** Kursat Ozdilli, Gozde Oztan, Yeliz Ogret, Rustu Oguz, Hayriye Senturk Ciftci, Filiz Aydın, Fatma Oguz

**Affiliations:** 1 Department of Medical Biology, Faculty of Medicine, Istanbul Medipol University, Istanbul 34810, Turkey; kozdilli@medipol.edu.tr; 2 Pediatric Bone Marrow Transplantation Unit, Medipol Mega University Hospital, Istanbul 34214, Turkey; 3 Department of Medical Biology, Istanbul Faculty of Medicine, Istanbul University, Istanbul 34093, Turkey; yelizd@istanbul.edu.tr (Y.O.); hayriye.senturkciftci@istanbul.edu.tr (H.S.C.); oguzsf@gmail.com (F.O.); 4 Department of Medical Biology, Faculty of Medicine, Demiroglu Bilim University, Istanbul 34394, Turkey; rusduoguz@gmail.com (R.O.); filiz.aydin@demiroglu.bilim.edu.tr (F.A.)

**Keywords:** macrophage migration inhibitory factor, *MIF*, rs755622, age-group comparison, older adults, promoter polymorphism, cross-sectional genetic association, eQTL

## Abstract

Age-associated immune–inflammatory remodeling may be influenced by regulatory variation in the macrophage migration inhibitory factor gene (*MIF*). We conducted an exploratory cross-sectional comparison to assess whether *MIF* rs755622 (−173G/C) genotype distributions differ between predefined younger and older age groups in a Turkish population and to characterize the observed pattern using genetic-model and in silico analyses. We evaluated 368 individuals: 245 older adults aged 65–102 years and 123 younger controls aged 20–46 years. None of the 26 main association tests remained statistically significant after global multiplicity correction (minimum FDR q = 0.062; minimum Bonferroni-adjusted *p* = 0.108). Before correction, GC frequency was higher in the older group and increased across the ordered age categories, and sex-adjusted analyses yielded concordant nominal estimates. However, these nominal patterns were sensitive to younger-control genotype reclassification and were not supported by an allele-level or additive association. Younger controls showed Hardy–Weinberg disequilibrium (*p* < 0.001), without sequencing confirmation, and deterministic and scenario-based Monte Carlo genotype-reclassification analyses indicated sensitivity of the nominal signal to uncertainty in control genotype classification. GTEx data provide C-allele-oriented expression context but do not validate function in this cohort. These preliminary findings warrant independent genotype verification, ancestry-matched replication, and direct functional investigation in future studies.

## 1. Introduction

Aging is a progressive and multifactorial biological process characterized by the gradual decline of physiological integrity, reduced adaptive capacity, and increased susceptibility to chronic diseases. Rather than representing a passive accumulation of damage, aging is now considered the result of interconnected molecular and cellular alterations, including genomic instability, telomere attrition, epigenetic remodeling, mitochondrial dysfunction, cellular senescence, stem-cell exhaustion, and altered intercellular communication [1]. These biological changes collectively influence both lifespan and healthspan and are shaped by genetic background, environmental exposures, and immune–inflammatory regulation [1,2]. Human longevity is therefore considered a complex polygenic phenotype in which immune regulation, inflammatory balance, metabolic adaptation, and stress-response pathways play central roles [2,3].

Among the biological processes involved in aging, chronic low-grade inflammation has received particular attention. This phenomenon, commonly referred to as inflammaging, is characterized by persistent, sterile, systemic inflammatory activation that increases with advancing age. Inflammaging contributes to several age-associated conditions, including cardiovascular disease, frailty, metabolic dysfunction, neurodegeneration, and cancer [4,5]. Closely related to inflammaging is immunosenescence, which refers to age-related remodeling and functional decline of both innate and adaptive immune responses. These two processes are biologically interconnected: immunosenescence may impair immune surveillance and pathogen control, whereas persistent inflammatory activation may accelerate tissue damage and age-related functional decline [6,7].

Macrophage migration inhibitory factor (MIF) is a pleiotropic pro-inflammatory cytokine with important roles in innate and adaptive immunity. MIF was initially described as a lymphocyte-derived factor that inhibits macrophage migration, but it is now recognized as an upstream regulator of inflammatory responses, leukocyte recruitment, cytokine production, glucocorticoid counter-regulation, cell survival, angiogenesis, and tumor biology [8,9,10,11]. MIF exerts its biological effects through CD74-mediated signaling and also functions as a non-cognate ligand for the chemokine receptors CXCR2 and CXCR4, thereby promoting ERK/MAPK activation, leukocyte recruitment, and inflammatory signaling [12,13]. Through these receptor systems, MIF activates downstream signaling pathways, including MAPK/ERK, PI3K/Akt, and NF-κB-associated inflammatory cascades, placing it at the interface of immune activation, cellular stress responses, and chronic inflammatory disease [8,12,13].

The human *MIF* gene is located on chromosome 22q11.23 and contains regulatory polymorphisms that may influence transcriptional activity and cytokine expression [14]. Among these, the *MIF* −173G/C (rs755622) promoter polymorphism has attracted interest because previous studies have reported regulatory associations at this locus. The substitution of guanine with cytosine at the historically designated −173 position has been reported to create a binding site for transcription factor activator protein-4 (AP-4), and the C allele has been associated with altered *MIF* promoter activity in several disease contexts [14,15,16]. In addition, the −794 CATT repeat polymorphism has been implicated in the regulation of *MIF* expression, supporting the need for broader promoter-level investigation [14,15].

Because aging is strongly influenced by inflammatory and immune-regulatory mechanisms, variants in cytokine genes may contribute to interindividual differences in age-associated phenotypes [3,4,5,6,7]. In this context, rs755622 is a biologically plausible candidate for age-group association studies because it is located in the promoter region of the *MIF* gene, which encodes an inflammatory mediator involved in immune-cell recruitment and vascular inflammatory processes [8,13,15,16].

Previous studies have linked *MIF* rs755622 to a broad spectrum of inflammatory, autoimmune, cardiovascular, fibrotic, and neoplastic conditions; however, the available evidence has largely focused on disease susceptibility rather than age-group status [17,18,19]. Despite the established involvement of MIF protein in inflammatory and immune-regulatory pathways, direct evidence linking the *MIF* −173G/C (rs755622) promoter variant to age-group status remains limited, and population-based studies evaluating its distribution across age strata, particularly among individuals aged ≥90 years, are scarce [17]. These limitations provided the rationale for examining whether rs755622 genotype distributions differ between younger and older individuals in a Turkish population.

Accordingly, the present study aimed to determine whether the *MIF* −173G/C (rs755622) promoter variant is associated with age-group status by comparing genotype and allele distributions between younger and older Turkish individuals, including a subgroup aged ≥90 years. We further aimed to characterize any observed signal using genetic inheritance models, sex-adjusted analyses, functional annotation, eQTL assessment, and a protein association network. We hypothesized that genotype distributions would differ across age groups without prespecifying the direction of an allele-specific effect. Age-group status constituted the study outcome; validated measures of healthy aging, healthspan, or inflammaging were not assessed. Accordingly, the cross-sectional design does not evaluate longitudinal aging trajectories, survival, lifespan, or clinically defined longevity.

## 2. Results

### 2.1. Characteristics of the Study Population

A total of 368 individuals were included, comprising 245 older adults aged 65–102 years and 123 younger controls aged 20–46 years. The mean age was 83.5 years in the older group and 43.0 years in the younger control group. The older cohort included 147 women and 98 men, whereas the younger control group included 55 women and 68 men.

For age-stratified analyses, the older cohort was divided into participants aged 65–89 years (*n* = 205) and those aged ≥90 years (*n* = 40). No participants aged 47–64 years were sampled. The demographic characteristics and age-group distribution are summarized in Table 1. Sex-specific sample sizes were available for the overall older and younger groups but not separately for the 65–89- and ≥90-year strata. Information on the exact recruitment settings and sample-collection periods was unavailable in the archived study records.

### 2.2. Genotype Distribution and Hardy–Weinberg Equilibrium

The analytical dataset contained genotype assignments for all 368 included participants. In the overall older group, the GG, GC, and CC genotypes were detected in 196 (80.0%), 44 (18.0%), and 5 (2.0%) individuals, respectively; the corresponding counts in younger controls were 108 (87.8%), 10 (8.1%), and 5 (4.1%). In the 65–89-year subgroup, genotype frequencies were 81.4%, 16.6%, and 2.0%, respectively, whereas the ≥90-year subgroup showed frequencies of 72.5%, 25.0%, and 2.5% (Table 2). The reported denominator therefore represents the analyzed cohort with complete genotype assignments; the analytical record does not enumerate pre-analysis failed or ambiguous attempts and is not interpreted as a laboratory call-rate denominator.

Exact Hardy–Weinberg equilibrium testing showed no significant deviation in the overall older group (*p* = 0.188), the 65–89-year subgroup (*p* = 0.238), or the ≥90-year subgroup (*p* = 1.000). In contrast, younger controls deviated significantly from equilibrium (*p* < 0.001). At the observed younger-control C-allele frequency of 8.13%, the expected genotype counts were 103.82 GG, 18.37 GC, and 0.81 CC, compared with the observed counts of 108, 10, and 5. Thus, the deviation comprised both a heterozygote deficit and an excess of CC calls. The source of this disequilibrium cannot be adjudicated from the aggregated genotype counts alone and was therefore examined through additional diagnostic and sensitivity analyses.

Because sex-stratified genotype counts were available for the overall younger and older groups, exact HWE was also evaluated within sex strata as a diagnostic analysis. Among younger controls, the deviation was evident in men (58 GG, 6 GC, and 4 CC; exact *p* = 0.00094) but not in women (50 GG, 4 GC, and 1 CC; exact *p* = 0.134). Neither older men (74 GG, 21 GC, and 3 CC; exact *p* = 0.382) nor older women (122 GG, 23 GC, and 2 CC; exact *p* = 0.342) showed significant disequilibrium. This stratification indicates that the overall control-group HWE signal was not distributed uniformly across sex strata, but it does not establish its underlying cause (Supplementary Table S7).

To provide descriptive population context, the younger-control GC frequency (8.1%) was compared with publicly available reference data. The 1000 Genomes Project Phase 3 data indicate GC heterozygote frequencies of approximately 31–33% across the EUR, EAS, and SAS superpopulations. The TSI (Toscani in Italy) population was additionally examined as a population-specific European benchmark, with a C-allele frequency of 18.2% [20]. gnomAD non-Finnish-European data indicate a GC frequency of approximately 31.7% and a C-allele frequency of approximately 19.0% [21]. These external benchmarks are not formal controls and cannot resolve the Hardy–Weinberg deviation because ancestry composition, recruitment, genotyping or sequencing methods, and quality-control procedures differ. The descriptive comparison is summarized in Supplementary Table S4.

Because independent re-genotyping or sequencing confirmation was not available, the source of the Hardy–Weinberg disequilibrium in younger controls remains unresolved. Statistical diagnostic and sensitivity analyses cannot substitute for laboratory-based genotype verification; therefore, association estimates involving the younger-control group should be regarded as provisional.

**Figure 1:**
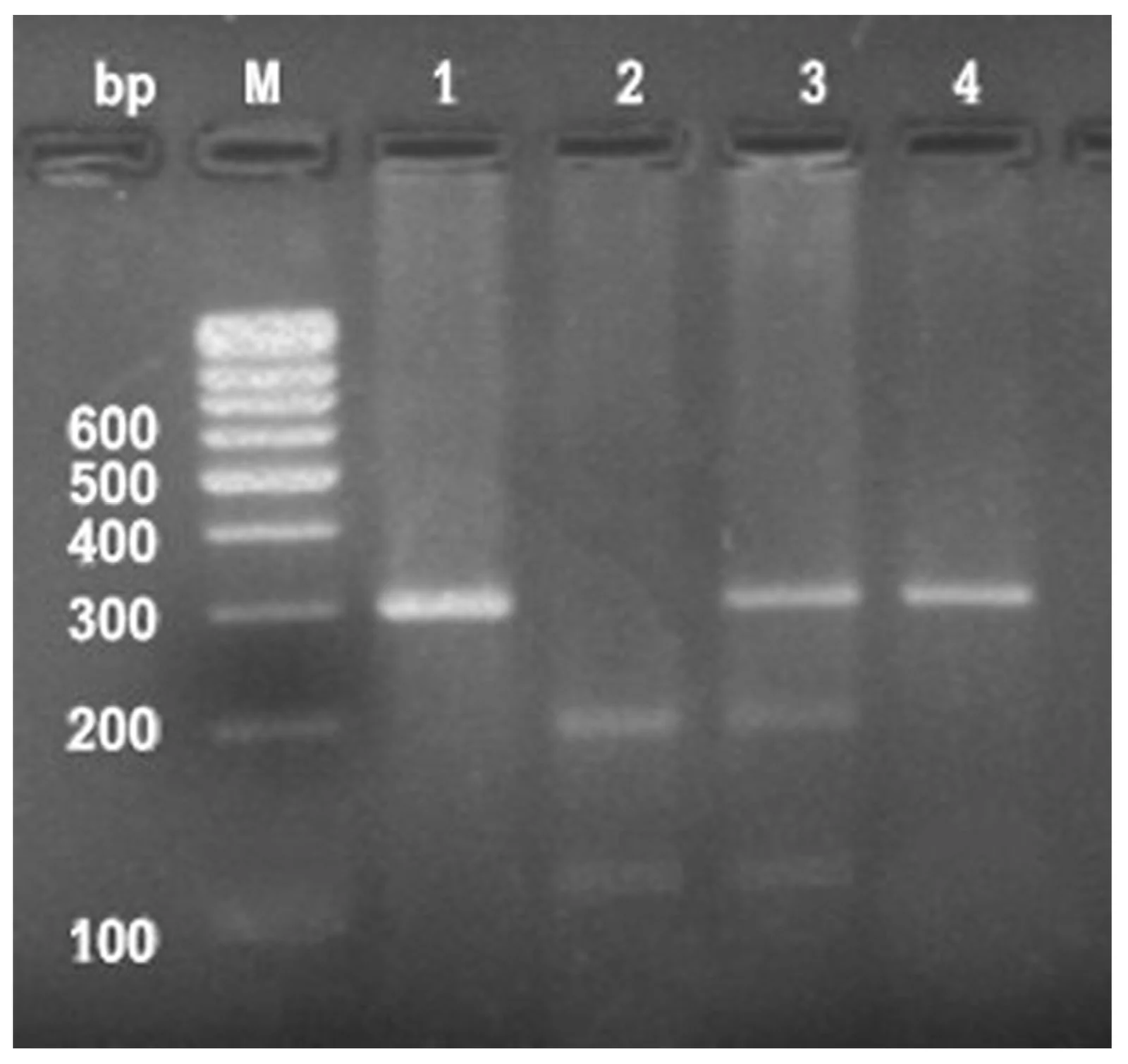
Representative PCR-RFLP electrophoresis profile of the *MIF* −173G/C (rs755622) polymorphism. M, 100 bp DNA ladder with molecular-size labels from 100 to 600 bp; lane 1, GG genotype (298 bp); lane 2, CC genotype (192 and 106 bp); lane 3, GC genotype (298, 192, and 106 bp); lane 4, undigested PCR-product control (298 bp). The image represents one electrophoresis run. The gel is presented as a representative illustration of the expected PCR-RFLP fragment patterns and does not constitute comprehensive run-level quality-control documentation or independent genotype verification.

Representative PCR-RFLP genotype patterns are shown in Figure 1.

### 2.3. Age-Stratified Genotype and Allele Association Analyses

The GC genotype was nominally more frequent in the overall older group than in younger controls (18.0% vs. 8.1%; OR = 2.474, 95% CI: 1.199–5.104; *p* = 0.012). In the mutually exclusive strata, GC frequency was 8.1% at ages 20–46 years, 16.6% at ages 65–89 years, and 25.0% at ages ≥90 years. An exploratory Cochran–Armitage test using ordered scores of 0, 1, and 2 yielded *Z* = 2.866 and a two-sided *p* = 0.004. An exact conditional permutation sensitivity test for the same ordered GC trend yielded a two-sided *p* = 0.0047, closely matching the asymptotic result. Because ages 47–64 years were not sampled and the categories are not equally spaced, these results represent an ordered-group trend rather than a linear age–dose relationship. Under the single global correction across 26 main association tests, the trend did not remain significant (FDR *q* = 0.062; Bonferroni-adjusted *p* = 0.108). The CC genotype remained uncommon, particularly in the ≥90-year subgroup, in which only one CC carrier was observed. The exact conditional result was treated as a re-estimation of the same trend hypothesis rather than as an additional main test (Supplementary Table S6).

**Figure 2:**
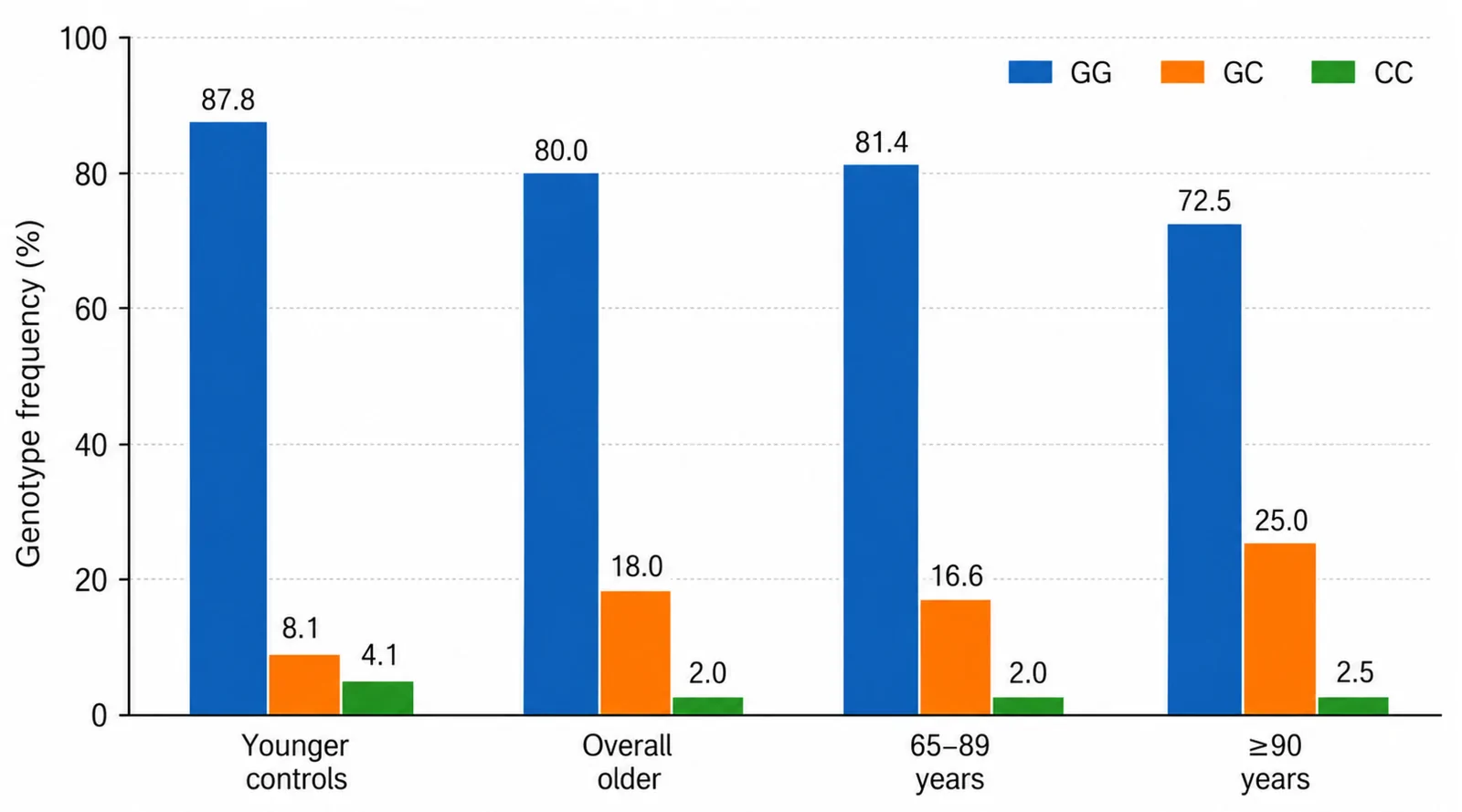
Genotype frequencies of the *MIF* rs755622 polymorphism across age groups. Percentages are based on group totals. The overall older group includes both the 65–89-year and ≥90-year subgroups and was not included as an additional category in the ordered trend test. No participants aged 47–64 years were sampled.

The C-allele frequency was 8.1% in younger controls, 11.0% in the overall older group, 10.2% in participants aged 65–89 years, and 15.0% in those aged ≥90 years. None of the allelic comparisons was nominally significant. A single global Benjamini–Hochberg correction was applied across all 26 main association tests, including genotype-specific, allelic, inheritance-model, sex-stratified, ordered-trend, and genotype-by-sex interaction analyses. No result remained significant (minimum *q* = 0.062), and no result survived global Bonferroni correction (minimum adjusted *p* = 0.108). The findings are therefore exploratory. Age-group estimates are summarized in Table 3, and genotype frequencies are visualized in Figure 2.

### 2.4. Genetic Inheritance Models and Sex-Adjusted Logistic Regression

No primary inheritance model was prespecified; accordingly, all model-specific analyses were interpreted as exploratory. The overdominant contrast (GC vs. GG + CC), which is algebraically equivalent to the unadjusted GC-versus-non-GC comparison in Table 3, was not considered independent evidence. Conventional logistic regression yielded an adjusted OR of 2.618 (95% CI: 1.259–5.444; *p* = 0.010), while the codominant GC-versus-GG comparison yielded an adjusted OR of 2.575 (95% CI: 1.237–5.363; *p* = 0.011). Under the global correction across 26 main association tests, both had FDR *q* = 0.062 and neither survived Bonferroni correction (adjusted *p* = 0.260 and 0.286, respectively). The dominant, recessive, and additive models were also non-significant after global correction (Table 4; Supplementary Table S2).

Firth penalized logistic regression was used as a sparse-data sensitivity analysis with adjustment for sex. The overdominant estimate was OR = 2.521 (95% CI: 1.222–5.200; *p* = 0.012), and the codominant GC-versus-GG estimate was OR = 2.480 (95% CI: 1.201–5.121; *p* = 0.014); the complete Firth results are reported in Supplementary Table S3. These analyses re-estimated the same six inheritance-model hypotheses and were therefore treated as sensitivity analyses rather than as six additional hypotheses in the global multiplicity family. The estimates were directionally similar to conventional logistic regression but do not resolve the control-group Hardy–Weinberg disequilibrium.

To reduce dependence on a particular inheritance model, a Fisher–Freeman–Halton exact test of the overall 2 × 3 genotype table yielded a nominal *p* = 0.022. A Cochran–Mantel–Haenszel analysis of the overdominant contrast stratified by sex produced a common OR of 2.606 (95% CI: 1.254–5.413; *p* = 0.008), with no evidence of heterogeneity between male and female strata (Breslow–Day *p* = 0.815). These model-free and stratified analyses re-expressed the same overall genotype and overdominant hypotheses, were not interpreted as independent evidence, and did not resolve the control-group HWE deviation (Supplementary Table S6).

### 2.5. Exploratory Sex-Stratified Analysis

Exploratory sex-stratified analyses provided no evidence of genotype-by-sex effect modification. The formal genotype-by-sex interaction under the overdominant model was non-significant (*p* = 0.815; global FDR q = 0.883). Complete sex-stratified genotype distributions and association estimates are provided in Supplementary Table S8 and are not interpreted as evidence of sex-specific association.

### 2.6. Deterministic and Scenario-Based Genotype-Reclassification Sensitivity Analyses

Because the younger controls showed a marked heterozygote deficit, a deterministic genotype-reclassification sensitivity analysis was performed under the overdominant model. Incrementally reclassifying younger-control participants from the non-GC category to the GC category attenuated the association: after two hypothetical reclassifications, the OR was 2.025 (*p* = 0.045), whereas after three reclassifications, the OR was 1.852 (*p* = 0.068). At eight hypothetical reclassifications, which approximately spans the observed heterozygote deficit relative to the HWE expectation, the OR was 1.277 (*p* = 0.463). Thus, the nominal association was sensitive to relatively small changes in younger-control genotype classification (Supplementary Table S1).

A scenario-based Monte Carlo genotype-reclassification sensitivity analysis was then used to summarize the same 0–8 younger-control reclassification range probabilistically. In 100,000 iterations, the number of potentially hidden GC calls among the observed younger-control non-GC genotypes was sampled with equal probability from 0 to 8 while the older-group counts remained fixed. The median simulated OR was 1.704, the 2.5th–97.5th percentile range of the simulated OR distribution was 1.277–2.474, the median two-sided Fisher p value was 0.129, and 33.3% of iterations retained nominal *p* < 0.05. Because the scenario distribution was not calibrated using independent genotype-validation data, these results quantify robustness under an explicit hypothetical reclassification model rather than estimating a corrected genotype effect (Supplementary Table S5). The loss of nominal significance after reclassification of only three of 123 younger controls demonstrates that the observed nominal association is not robust to small plausible changes in control genotype classification.

### 2.7. External Functional and Regulatory Annotation of the MIF rs755622 Locus

Ensembl Variant Effect Predictor annotation mapped rs755622 to chromosome 22 at GRCh38 position chr22:23,894,205, with a G>C substitution in the non-coding 5′ promoter/regulatory region of the *MIF* locus. Although historically designated as −173G/C, the current RefSeq transcript nomenclature places the variant 270 bases upstream of the transcription start site (NM_002415.2:c.−270G>C). No protein-coding or amino-acid-altering consequence was identified. This annotation defines the genomic context of the variant but does not by itself demonstrate regulatory function [22,23]. The principal annotation features are summarized in Table 5.

Published ENCODE-informed chromatin mapping provides complementary locus-level regulatory context. In the linkage-disequilibrium region containing rs755622 and the *MIF* promoter, both H3K4me1 and H3K27ac marks were reported in primary neutrophils and CD4+ T cells; the neutrophil locus also contained multiple potential transcription-factor-binding sites derived from ENCODE ChIP-seq tracks [24]. These features are compatible with an active regulatory chromatin environment at the broader locus but do not identify rs755622 itself as the causal regulatory nucleotide or establish an allele-specific effect in this cohort.

### 2.8. External GTEx eQTL Context for rs755622 and MIF Expression

GTEx v8-derived analysis showed associations between rs755622 and variation in *MIF* expression in several non-central-nervous-system tissues. In GTEx single-tissue eQTL reporting, the normalized effect size is oriented to the alternate allele. Because rs755622 is represented as G>C on GRCh38, the C allele is the effect allele for the values reported here. The largest selected positive NES was observed in the esophagus–gastroesophageal junction (NES = 0.482; *p* = 1.2 × 10^−7^), with additional positive associations in coronary artery, aorta, cultured fibroblasts, and tibial artery [25,26].

In tissues relevant to systemic inflammation and aging, the C allele was associated with higher *MIF* expression in whole blood (NES = 0.319; *p* = 1.4 × 10^−6^), lung (NES = 0.263; *p* = 6.7 × 10^−4^), and spleen (NES = 0.220; *p* = 0.040). The testis result was excluded because the nominal p value and m value in the source summary could not be reconciled. Table 6 therefore includes only tissues with internally consistent single-tissue summary statistics and reports sample size, C-allele-oriented NES, and nominal p value.

**Figure 3:**
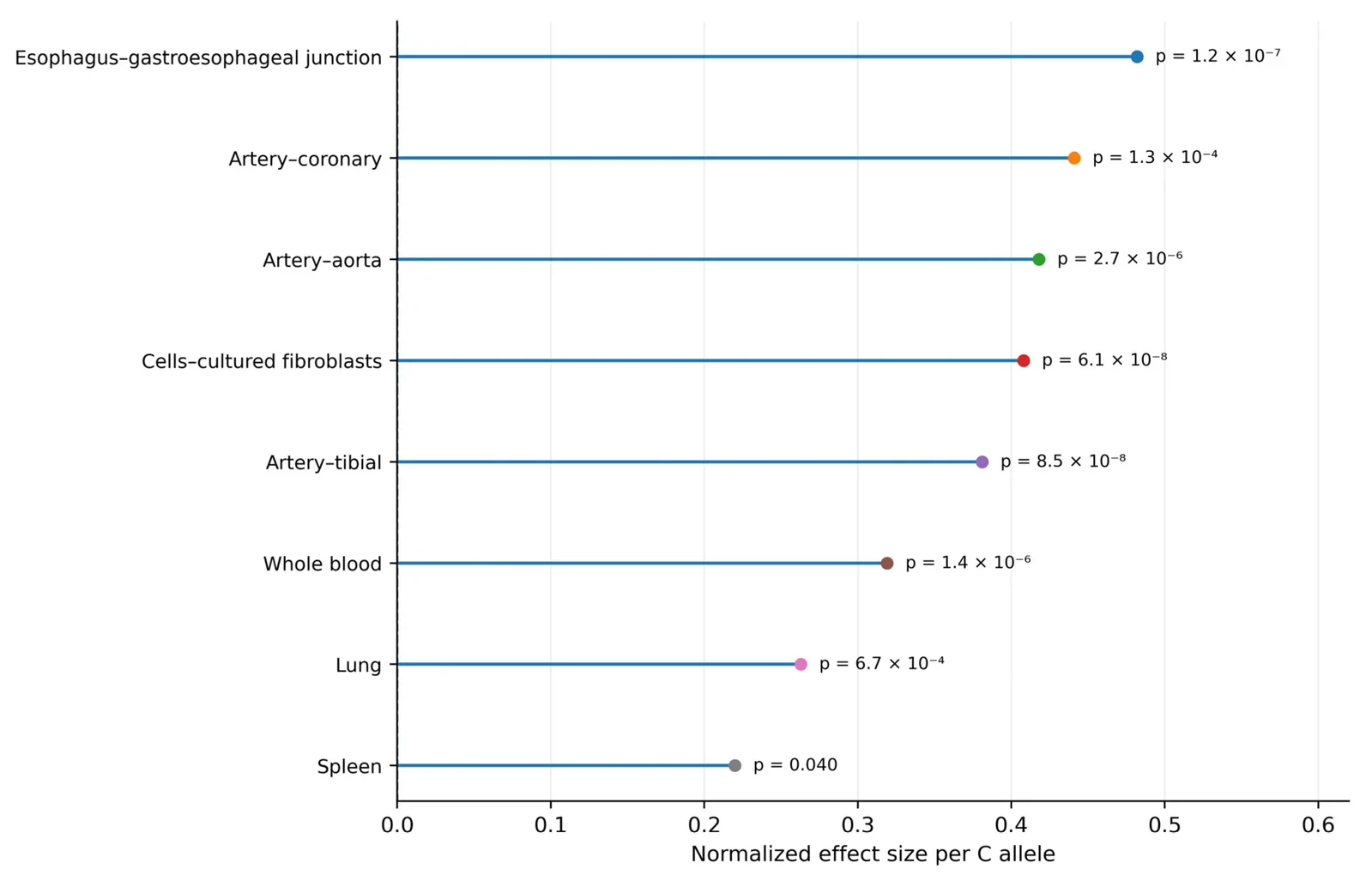
Effect-size lollipop plot of selected GTEx v8 single-tissue eQTL associations between rs755622 and MIF expression. The C allele represents the alternate/effect allele; positive normalized effect size (NES) values indicate higher normalized MIF expression per C allele. Points indicate tissue-specific NES estimates, with horizontal stems extending from NES = 0 to each estimate.

These external data are consistent with tissue-dependent C-allele associations with *MIF* expression, particularly in blood and vascular tissues. However, GTEx v8 comprised predominantly European American donors (85.3%), and donor ages were limited to approximately 21–70 years; therefore, the dataset differs from the present Turkish cohort and does not validate an expression effect in individuals aged ≥90 years. Selected C-allele-oriented effect sizes are visualized in Figure 3.

### 2.9. MIF-Centered Protein Association Network

STRING analysis generated an MIF-centered protein association network containing 11 nodes and 31 undirected associations, with a mean node degree of 5.64. MIF was the central node and was associated with all 10 first-shell proteins included in the network. The highest-scoring MIF-associated proteins were CD74 (combined STRING score = 0.999), CXCR2 (0.993), CXCR4 (0.991), and COPS5 (0.976). The principal MIF-associated proteins, STRING combined scores, node degrees, and functional contexts are summarized in Table 7. CD74, CXCR2, and CXCR4 are established components of MIF-related receptor and chemotactic signaling, whereas COPS5 links the network to protein-turnover and cellular-stress pathways.

**Figure 4:**
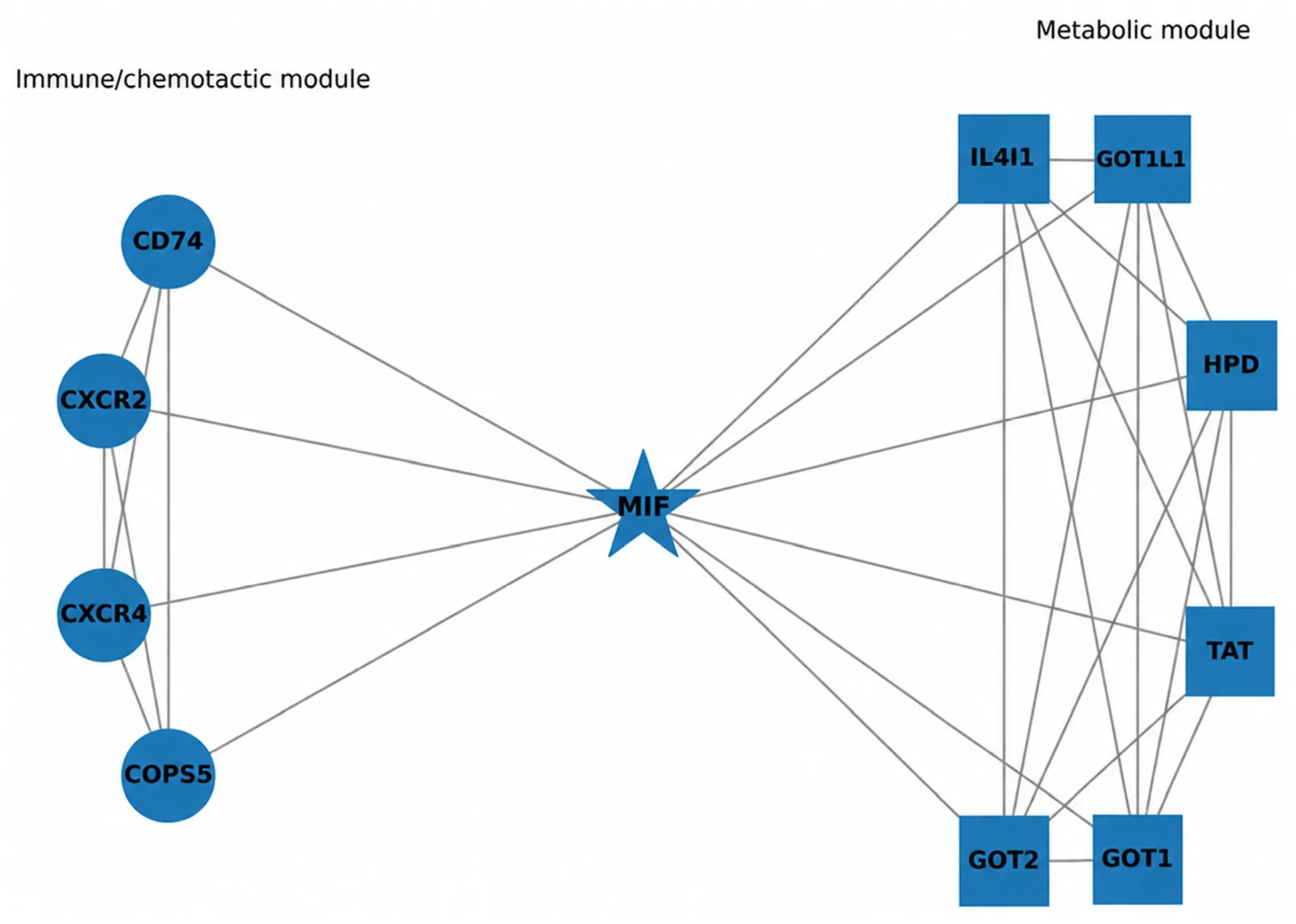
MIF-centered protein association network generated from STRING v12.0 using a medium-confidence interaction score threshold of 0.400, with 10 first-shell interactors and no second-shell interactors. Circles represent proteins primarily associated with immune/chemotactic functions, whereas squares represent proteins associated with metabolic functions; MIF is shown as the central seed protein. Connecting lines represent STRING-supported functional or physical associations among the proteins.

Additional high-scoring associations included GOT2, GOT1, TAT, HPD, GOT1L1, and IL4I1, indicating a broader metabolic component involving amino-acid metabolism and antigen-related processes. Because STRING integrates both physical and indirect functional associations, these edges should not uniformly be interpreted as direct protein binding [27]. The reconstructed MIF-centered network is shown in Figure 4.

## 3. Discussion

The present study investigated the association between the *MIF* promoter polymorphism rs755622 and age-group status in a Turkish population. Importantly, the study compares predefined age groups and should not be interpreted as a longitudinal investigation of aging, survival, or longevity. GC frequency increased across the mutually exclusive age categories, and the ordered-group trend was nominally significant. Additional model-free exact, exact conditional trend, and sex-stratified Cochran–Mantel–Haenszel sensitivity analyses were nominally concordant; however, they re-expressed the same underlying genotype counts and did not constitute independent replication. The trend, the genotype-specific comparisons, the sex-adjusted inheritance-model results, the sex-stratified comparisons, and the interaction test were evaluated together in a single global family of 26 main association tests. None remained significant after Benjamini–Hochberg or Bonferroni correction. The minimum global FDR value was *q* = 0.062. Accordingly, the evidence should be regarded as exploratory and correction-sensitive.

The concentration of the nominal signal in GC carriers and the absence of a corresponding allele-dose association require particular caution. The overdominant contrast is not independent of the unadjusted GC-versus-non-GC comparison and does not establish biological heterozygote advantage. Accordingly, neither the G nor the C allele should be described as a risk or protective allele on the basis of these data. Allele-specific transcription, non-linear promoter activity, linkage with other *MIF* promoter variants, sampling structure, and genotype-classification uncertainty could each contribute to a GC-centered pattern. The deterministic reclassification analysis showed that moving only three younger controls from non-GC to GC attenuated the OR to 1.852 (*p* = 0.068), and the scenario-based Monte Carlo analysis yielded a median OR of 1.704 with a median Fisher p value of 0.129; only 33.3% of iterations retained nominal *p* < 0.05. Thus, the nominal GC-centered signal depends materially on the original classification of a very small number of younger controls and should not be interpreted as stable evidence of association in the absence of independent genotype verification. These sensitivity analyses quantify the fragility of the association under explicit reclassification scenarios and do not represent corrected genotype calls.

The biological rationale for investigating rs755622 in relation to age-group status is supported by the established involvement of MIF in immune regulation and chronic inflammation. Aging is accompanied by inflammaging and immunosenescence, which together produce persistent low-grade inflammatory activation, impaired immune surveillance, altered leukocyte function, and progressive tissue dysfunction [4,5,6,7]. MIF is an upstream regulator of innate and adaptive immune responses and can promote leukocyte recruitment, cytokine production, glucocorticoid counter-regulation, cell survival, and stress adaptation [8,9,10]. It exerts extracellular effects through receptor complexes involving CD74 and CD44 and functions as a non-cognate ligand of the chemokine receptors CXCR2 and CXCR4 [12,13,28]. These receptor systems activate downstream pathways including MAPK/ERK, PI3K/Akt, AMPK, and other inflammatory and stress-response cascades. More broadly, recent experimental evidence from hepatocellular carcinoma has further demonstrated the context-dependent involvement of PI3K/AKT/mTOR signaling in the effects of upstream regulatory proteins [29]. MIF is therefore biologically positioned at the interface of inflammation, immune-cell trafficking, vascular homeostasis, metabolism, and tissue adaptation, all of which are relevant to aging biology [13].

Experimental studies of longevity-associated genes, such as *Cisd2*, have demonstrated that genetic regulation of mitochondrial homeostasis can influence lifespan and delay age-related tissue deterioration in animal models [30]. Nevertheless, aging is not governed by a single gene or biological pathway but instead emerges from complex interactions among mitochondrial, metabolic, inflammatory, immune, and stress-response mechanisms. In this context, the nominal genotype pattern observed in the present study provides a hypothesis-generating basis for further investigation of *MIF* within the broader network of biological pathways involved in aging.

The functional annotation findings provide genomic context for interpreting the genetic results. Ensembl VEP classified rs755622 as a non-coding variant in the upstream promoter/regulatory region of the *MIF* locus, without a predicted protein-coding or amino-acid-altering consequence [22]. Complementary ENCODE-informed chromatin mapping of the rs755622-containing linkage-disequilibrium region in primary neutrophils and CD4+ T cells identified H3K4me1 and H3K27ac marks, while the neutrophil region contained multiple potential transcription-factor-binding sites [24]. Together, these observations support a regulatory chromatin environment surrounding the *MIF* promoter. Because the chromatin evidence is locus-level and cell-context dependent rather than an allele-specific assay of rs755622, cohort-specific molecular effects remain to be established by direct expression, protein, or promoter-level measurements.

The GTEx-derived analysis identified tissue-dependent associations between rs755622 and *MIF* expression in several tissues relevant to systemic inflammation and vascular biology. GTEx single-tissue NES values are oriented to the alternate C allele; positive values therefore indicate higher normalized *MIF* expression per C allele. This direction makes the absence of a cohort-level allelic association and additive dose response more difficult to reconcile with a simple transcriptional mechanism. Moreover, GTEx donors were predominantly European American and no older than approximately 70 years, so these data cannot establish an expression effect in the Turkish participants or in the ≥90-year subgroup [25,26].

Additional biological context was provided by the MIF-centered STRING network. The principal MIF-associated proteins included CD74, CXCR2, CXCR4, and COPS5, together with proteins involved in amino-acid metabolism and cellular metabolic homeostasis, including GOT1, GOT2, TAT, HPD, GOT1L1, and IL4I1. The prominent positions of CD74, CXCR2, and CXCR4 are consistent with the known extracellular functions of MIF in antigen-related signaling, leukocyte chemotaxis, immune-cell recruitment, and inflammatory activation [12,13,28]. The association with COPS5, also known as JAB1, provides a connection with intracellular stress signaling, protein turnover, and regulation of transcriptional responses. The presence of aminotransferases and amino-acid-processing enzymes suggests that the broader MIF-associated network may also intersect with metabolic pathways relevant to cellular homeostasis. However, STRING integrates both physical interactions and indirect functional associations; accordingly, the network does not demonstrate that all identified proteins directly bind MIF or that rs755622 modifies any of these interactions [27].

The nominal genotype-level pattern observed in the present study can be considered in the context of previous evidence that rs755622 may have phenotype-dependent immunological associations. A global meta-analysis reported heterogeneous associations across the disease spectrum, with stronger evidence in autoimmune and infectious conditions than in several age-related disease categories [17]. Associations between rs755622 and cancer susceptibility have also been reported, although the strength and direction of these associations have varied among malignancies and populations [18]. In Chinese Han individuals, rs755622 was associated with acute coronary syndrome, supporting a potential role in vascular inflammatory phenotypes [19]. Lin et al. identified an association between *MIF* polymorphisms and breast cancer susceptibility in Chinese women, whereas Brill et al. reported that high-expression genotypes were related to neuromyelitis optica disease severity rather than disease occurrence [31,32]. These findings collectively suggest that the phenotypic consequences of rs755622 are unlikely to be uniform and may depend on ancestry, disease context, age, sex, environmental exposures, and interactions with other regulatory variants [17].

The study by Alban et al. is particularly relevant to the genotype-specific pattern observed here. In glioblastoma cohorts, carriers of the minor allele, predominantly individuals with the GC genotype, exhibited differences in immune-cell composition and increased expression of immune-related signatures, although the polymorphism was not associated with disease incidence, survival, or a consistent difference in overall *MIF* expression [26]. This indicates that rs755622 may be associated with context-dependent immunological phenotypes without necessarily producing a simple or uniform change in *MIF* transcript abundance. Although glioblastoma and aging are biologically distinct conditions, these observations support the broader interpretation that rs755622 may influence immune regulation in a genotype- and tissue-dependent manner. They do not, however, establish a causal relationship with longevity [26].

Experimental studies similarly indicate that the contribution of MIF to aging is complex and potentially bidirectional. Harper et al. reported that *MIF*-deficient mice were long-lived and retained a lifespan response to caloric restriction, suggesting that chronic MIF activity may, under some conditions, contribute to inflammatory or neoplastic processes that limit survival [33]. In contrast, Xu et al. demonstrated that *MIF* deficiency aggravated age-related cardiac remodeling and dysfunction despite reducing cardiac inflammation, potentially through disruption of autophagy and cellular energy homeostasis [34]. These apparently divergent observations indicate that MIF cannot be classified solely as detrimental or protective in aging. Its effects may vary according to tissue, inflammatory intensity, metabolic state, age, and disease context. Accordingly, the higher frequency of the GC genotype in the older groups should not be interpreted as evidence that increased or decreased MIF activity is universally advantageous [33].

Sex-stratified analyses provided no evidence of effect modification by sex. The formal genotype-by-sex interaction was non-significant (*p* = 0.815; global FDR q = 0.883); therefore, nominal subgroup differences were not interpreted as sex-specific associations. The present study has several strengths. It evaluated a relatively large group of older individuals and included an age-extreme subgroup aged ≥90 years, allowing genotype distributions to be examined across progressively older age categories. The cohort-level genetic observations were triangulated with external variant annotation, ENCODE-informed regulatory chromatin context, tissue-specific eQTL data, and an MIF-centered protein association network. This framework broadens biological interpretation beyond genotype-frequency comparisons while maintaining a clear distinction between external functional context and cohort-specific molecular validation.

Interpretation should be framed within the design and sampling structure of the study. The analysis is cross-sectional rather than prospective, and the 47–64-year interval was not sampled; accordingly, the Cochran–Armitage result represents an ordered-group comparison rather than a continuous age effect. The archived dataset supports age, sex, genotype, and consent variables but not standardized frailty, activities-of-daily-living, comorbidity, inflammatory-biomarker, or immune-cell phenotyping. Detailed recruitment-setting and sample-collection-period metadata are also outside the available analytical dataset, and sex-specific counts are not available separately for the 65–89-year and ≥90-year strata. The study outcome is therefore age-group membership rather than a clinically validated healthy-aging phenotype, and survival selection, birth-cohort differences, environmental exposures, and population structure remain relevant alternative explanations.

The analytical scope is also defined by the available molecular and genetic variables. The ≥90-year subgroup is relatively small and the CC genotype is rare, resulting in imprecise estimates. The minimum-detectable-effect calculation is descriptive and should not be interpreted as evidence that the observed association was adequately powered or robust. The estimated 80% power threshold (OR ≈ 2.56) is close to the observed nominal ORs (approximately 2.47–2.62), indicating that the study had limited sensitivity to detect more modest genetic effects. Accordingly, the proximity of the observed estimates to the minimum detectable effect does not constitute independent support for the association. The cohort dataset is centered on rs755622, so within-cohort haplotype structure with the −794 CATT repeat and marker-based ancestry adjustment require multilocus genotype information beyond the present dataset. Population structure was therefore contextualized descriptively using external reference frequencies rather than treating those benchmarks as formal controls. Functional interpretation was triangulated across VEP, ENCODE-informed chromatin context, GTEx, and STRING, whereas cohort-specific molecular effects remain to be established by direct expression, protein, or promoter-level assessment. Because the cohort was genotyped only for rs755622, lacked −794 CATT repeat data and marker-based ancestry information, and had no within-cohort MIF expression or protein measurements, the present study cannot distinguish a direct functional effect of rs755622 from linkage with another regulatory variant or from an association attributable to population stratification. Accordingly, the external VEP, ENCODE, GTEx, and STRING analyses provide biological context but do not constitute causal validation of rs755622. None of the 26 main association tests survived global FDR or Bonferroni correction.

The younger-control Hardy–Weinberg equilibrium result warrants particular attention. At the observed C-allele frequency, 103.82 GG, 18.37 GC, and 0.81 CC genotypes were expected, compared with 108, 10, and 5 observed. The deviation therefore combines a heterozygote deficit with CC excess. Sex-stratified exact HWE analysis further showed that the deviation was concentrated in younger men (*p* = 0.00094), whereas younger women did not deviate significantly (*p* = 0.134); older male and female strata also showed no significant disequilibrium. This diagnostic localization narrows the pattern but does not identify its source. Deterministic and scenario-based Monte Carlo reclassification analyses showed that modest changes in younger-control classification materially attenuate the nominal association, while Fisher–Freeman–Halton, exact-trend, Cochran–Mantel–Haenszel, and Firth analyses all rely on the same underlying genotype calls. The younger-control GC frequency of 8.1% was substantially lower than the approximately 31–33% heterozygote frequencies reported for the 1000 Genomes Phase 3 EUR, EAS, and SAS superpopulations and the approximately 31.7% value in gnomAD non-Finnish-European data [20,21]. The younger-control C-allele frequency of 8.1% was also lower than the 18.2% frequency observed in the TSI (Toscani in Italy) population, the approximately 19.0% gnomAD non-Finnish-European estimate, and the 24.6% and 18.7% values reported in two Turkish control cohorts [20,21,35,36]. The source of the control-group disequilibrium cannot be adjudicated by statistical re-analysis alone, and independent genotype verification remains necessary to distinguish classification uncertainty from sampling, batch, or population-structure explanations. Accordingly, until the younger-control genotypes are independently verified, the nominal association cannot be regarded as a robust genetic association.

Accordingly, the observed pattern is best viewed as hypothesis-generating evidence about age-group membership in this sampled cohort. Independent genotype verification and ancestry-matched replication would be required before extending the finding to causal longevity, clinically validated healthspan, inflammaging, or an allele-specific functional mechanism.

## 4. Materials and Methods

### 4.1. Study Design and Participants

This cross-sectional genetic association study investigated the relationship between the MIF promoter polymorphism −173G/C (rs755622) and age-group status in a Turkish population. Genotype and allele distributions were compared between younger and older participants, and the older cohort was stratified into predefined age groups. Age-group status, rather than a validated healthy-aging or inflammaging phenotype, was the study outcome. No primary inheritance model was prespecified; accordingly, all model-specific analyses were interpreted as exploratory. Reporting considerations for genetic association studies were informed by STREGA [37].

A total of 368 unrelated Turkish individuals were included: 245 older adults aged 65–102 years and 123 younger controls aged 20–46 years. The older cohort comprised 205 participants aged 65–89 years and 40 aged ≥90 years. No participants aged 47–64 years were sampled. Age and sex were recorded for all participants. The older cohort included 147 women and 98 men, whereas the younger group included 55 women and 68 men. Sex-specific counts for the 65–89-year and ≥90-year strata were unavailable.

Participants were eligible if they were of Turkish ancestry, had an available peripheral-blood sample suitable for DNA extraction, had complete age and sex information, and had provided written informed consent. Participants with insufficient DNA quantity or quality, incomplete demographic information, or unsuccessful genotype assignment were excluded from the analytical dataset. The archived study records did not contain standardized clinical phenotyping or detailed information on recruitment settings and sample-collection periods. The study was conducted in accordance with the Declaration of Helsinki and approved by the Ethics Committee of Haliç University at its meeting held on 17 March 2011 (meeting no. 2011-03). All participants provided written informed consent before enrollment.

### 4.2. DNA Isolation and Genotyping

Peripheral venous blood samples were collected into K2EDTA-containing tubes (Becton, Dickinson and Company, Franklin Lakes, NJ, USA). Genomic DNA was isolated from peripheral-blood leukocytes using the DTAB/CTAB extraction method with dodecyltrimethylammonium bromide (DTAB) and cetyltrimethylammonium bromide (CTAB) (Sigma-Aldrich, St. Louis, MO, USA), and purified DNA was stored at −20 °C until genotyping. Genotyping of the MIF −173G/C (rs755622) promoter polymorphism was performed using polymerase chain reaction–restriction fragment length polymorphism (PCR-RFLP). Custom oligonucleotide primers were synthesized by Integrated DNA Technologies (Coralville, IA, USA). The forward primer was 5′-ACT AAG AAA GAC CCG ACG C-3′ and the reverse primer was 5′-GGG GCA CGT TGG TGT TTA C-3′, generating a 298 bp PCR product encompassing the polymorphic site.

Following amplification, 5 µL of PCR product was digested in a final reaction volume of 10 µL containing 1 µL of 10× FastDigest Buffer, 0.5 µL of FastDigest MboI (Thermo Scientific, Thermo Fisher Scientific, Waltham, MA, USA; 0.5 FastDigest unit [FDU]), and 3.5 µL of nuclease-free water (Thermo Fisher Scientific, Waltham, MA, USA). Restriction digestion was performed at 37 °C for 15 min. Digestion products were separated on a 4% molecular-biology-grade agarose gel (Sigma-Aldrich, St. Louis, MO, USA), stained with ethidium bromide (Sigma-Aldrich, St. Louis, MO, USA), and visualized under ultraviolet illumination using a Gel Doc™ XR+ Gel Documentation System (Bio-Rad Laboratories, Hercules, CA, USA). A GeneRuler™ 100 bp DNA Ladder (Thermo Fisher Scientific, Waltham, MA, USA) was used as the molecular-size marker.

Genotypes were assigned according to the complete expected restriction-fragment pattern: GG, 298 bp; CC, 192 and 106 bp; and GC, 298, 192, and 106 bp. Because the 106 bp fragment is relatively small, electrophoretic resolution was assessed relative to the 100 bp molecular-size ladder, and genotype assignment was based on the complete fragment pattern rather than on the presence of the 106 bp fragment alone. Figure 1 shows the 100 bp DNA ladder, representative GG, CC, and GC patterns, and an undigested 298 bp PCR-product control.

Quality-control assessment was based on the available archived study records. Genotype assignment relied on the complete expected restriction-fragment pattern, and only participants with successful genotype assignments were included in the analytical dataset. The archived records did not provide a complete sample-level log of failed or initially ambiguous genotype calls; therefore, the number and proportion of such calls could not be reconstructed retrospectively. Likewise, run-level positive/negative control records, replicate-genotyping and duplicate-concordance data, and electrophoresis voltage and run duration were not available in the archived documentation. Independent genotype verification using an alternative genotyping method or sequencing was not available for the archived samples. The representative gel shown in Figure 1 includes an undigested 298 bp PCR-product control together with the expected genotype-specific restriction patterns and is presented as an illustrative example rather than as comprehensive run-level quality-control documentation. Accordingly, the reported quality-control information is restricted to procedures and controls that could be verified from the available study records.

### 4.3. Statistical Analysis

Statistical analyses were performed using SPSS version 14.0 (SPSS Inc., Chicago, IL, USA). Additional statistical analyses were performed using Python version 3.13.5 (Python Software Foundation), SciPy version 1.17.0 (SciPy community), and statsmodels version 0.14.6 (statsmodels developers).

The distribution of genotypes was evaluated for deviation from Hardy–Weinberg equilibrium using Arlequin version 3.5.2.2 (Computational and Molecular Population Genetics Laboratory, University of Bern, Bern, Switzerland) [38]. Differences in genotype and allele frequencies were assessed using Pearson’s chi-square test or Fisher’s exact test, as appropriate. Odds ratios with 95% confidence intervals were calculated to estimate associations with age-group status. Statistical significance was defined as a two-sided *p* value < 0.05 before multiplicity correction. As a diagnostic follow-up to the younger-control HWE result, exact HWE tests were additionally calculated within male and female strata for the younger and overall older groups using the observed sex-specific genotype counts. These stratified HWE tests assessed genotype-distribution quality rather than association hypotheses and were not included in the 26-test multiplicity family.

As additional model-free and stratified sensitivity analyses, the overall 2 × 3 table of age-group status by GG/GC/CC genotype was evaluated using the Fisher–Freeman–Halton exact test. The overdominant contrast was also evaluated using a Cochran–Mantel–Haenszel common odds ratio stratified by sex, and homogeneity of the stratum-specific odds ratios was assessed using the Breslow–Day test. An exact conditional permutation test re-evaluated the ordered GC trend while conditioning on the group sizes and the total number of GC carriers. These procedures were implemented in Python using exact enumeration and statsmodels and were treated as alternative re-estimations of existing hypotheses rather than as additional main tests.

Only rs755622 was genotyped in the cohort. The available dataset therefore supports single-variant association analyses, whereas within-cohort linkage-disequilibrium/haplotype estimation with the −794 CATT repeat and principal-component or other marker-based population-structure adjustment require multilocus genotype information. Population structure was contextualized descriptively using external reference-population frequencies rather than treating these benchmarks as formal controls. Accordingly, the available cohort data do not permit separation of a direct rs755622 effect from linkage with other MIF promoter variants or from effects attributable to population structure.

To comprehensively evaluate the genetic association, genotype distributions were additionally analyzed under codominant, dominant, recessive, overdominant, additive, and allelic inheritance models. In the codominant model, the GC and CC genotypes were compared separately with the GG reference genotype. In the dominant model, carriers of at least one C allele (GC + CC) were compared with GG homozygotes. In the recessive model, CC homozygotes were compared with carriers of the G allele (GG + GC). In the overdominant model, heterozygous individuals carrying the GC genotype were compared with participants carrying the GG or CC genotypes (GC vs. GG + CC). In the additive model, genotypes were coded according to the number of C alleles as 0, 1, and 2 for GG, GC, and CC, respectively. In the allelic model, G- and C-allele frequencies were compared between the study groups. These inheritance-model analyses were performed for the overall older-versus-younger comparison. Age-stratified analyses were limited to genotype-specific and allelic comparisons between younger controls and participants aged 65–89 years and ≥90 years.

A descriptive minimum-detectable-effect calculation estimated the odds ratio required for approximately 80% power using the current group sizes and the observed younger-control GC frequency. This calculation was used to characterize the detectable effect-size range of the study and not as post hoc evidence supporting the observed association. Conventional binary logistic regression used age-group status as the dependent variable, genotype status as the independent variable, and sex as an adjustment variable. Given sparse CC cells, Firth penalized logistic regression with sex adjustment was performed as a sensitivity analysis using Jeffreys-prior penalized likelihood; Wald confidence intervals were calculated from the inverse penalized information matrix. A genotype-by-sex interaction term was evaluated under the overdominant model, and sex-stratified genotype comparisons were treated as exploratory subgroup analyses.

Because younger controls deviated from HWE, two complementary genotype-reclassification sensitivity analyses were conducted under the overdominant contrast. First, a deterministic analysis incrementally reclassified 0–8 younger-control participants from the non-GC category to GC while the older-group counts remained fixed. Second, a scenario-based Monte Carlo analysis used 100,000 iterations following general principles of probabilistic sensitivity analysis [39]. The scenario parameter K, representing potentially hidden GC calls among observed younger-control non-GC genotypes, was sampled from a discrete uniform distribution over the integers 0–8. This range extends from no reclassification to approximately the observed heterozygote deficit relative to the HWE expectation (18.37 expected GC versus 10 observed). For each iteration, older-group counts were held at 44 GC and 201 non-GC and younger-control counts were set to 10 + K GC and 113 − K non-GC. The cross-product OR and two-sided Fisher exact p value were calculated for each iteration using random seed 20260807. The simulated distribution was interpreted as a scenario-based robustness distribution rather than as a corrected estimate of the true genotype effect. These genotype-reclassification sensitivity analyses were designed to quantify the potential impact of genotype-classification uncertainty on the association estimates and do not provide independent verification of the original genotype assignments.

A Cochran–Armitage ordered-group trend test compared GC status across the mutually exclusive age categories 20–46, 65–89, and ≥90 years using scores 0, 1, and 2. The overlapping overall older group was not entered as a fourth trend category. Because ages 47–64 years were absent and category widths differed, the test was interpreted as an ordered-group trend and not as a continuous linear age effect.

To avoid conflicting correction families for overlapping contrasts, a single global multiplicity family was used for 26 main association tests: 12 age-group genotype-specific or allelic comparisons, six sex-adjusted inheritance-model comparisons, six sex-stratified genotype comparisons, one Cochran–Armitage trend test, and one genotype-by-sex interaction test. The six Firth analyses were sparse-data sensitivity re-estimates of the same inheritance-model hypotheses and were not counted as additional tests. The deterministic and scenario-based Monte Carlo genotype-reclassification analyses and the additional Fisher–Freeman–Halton, exact conditional trend, Cochran–Mantel–Haenszel, and Breslow–Day analyses were also treated as sensitivity re-expressions of existing hypotheses rather than additional main tests. Benjamini–Hochberg FDR correction was the primary adjustment, and global Bonferroni correction was reported as a stringent sensitivity analysis. Multiplicity-adjusted values were calculated from unrounded *p* values; values displayed in the tables were rounded for presentation. No association met an adjusted threshold of 0.05.

### 4.4. In Silico Functional Annotation, Regulatory Chromatin Context, eQTL, and Protein Association Network Analyses

The bioinformatic context of the *MIF* promoter polymorphism rs755622 was evaluated using complementary in silico variant annotation, regulatory-chromatin context, expression quantitative trait locus, and protein-association-network approaches. Variant annotation was performed using the Ensembl Variant Effect Predictor (VEP; Ensembl release 116, GRCh38.p14 reference assembly; Ensembl, EMBL-EBI). The genomic position, allelic substitution, relationship with the *MIF* transcript, sequence consequence, and regulatory-region annotation were recorded. The nomenclature NM_002415.2:c.−270G>C (historically −173G/C) was used where appropriate [22,23]. VEP annotation was interpreted as genomic-context information rather than experimental evidence of regulatory function.

Regulatory chromatin context was additionally reviewed using published ENCODE-informed epigenomic mapping of the rs755622-containing linkage-disequilibrium region in primary neutrophils and CD4+ T cells. H3K4me1 and H3K27ac chromatin marks and transcription-factor-binding features within the broader locus were recorded as external evidence of regulatory chromatin activity [24]. This locus-level evidence was not interpreted as proof of allele-specific activity of rs755622.

For descriptive population context, rs755622 genotype and allele frequencies were reviewed in the 1000 Genomes Project Phase 3 EUR, EAS, and SAS superpopulations and in the gnomAD v2.1.1 non-Finnish-European reference population, while the C-allele frequency in TSI (Toscani in Italy) was additionally examined as a population-specific European benchmark [20,21]. These public frequencies were used only as external benchmarks; they were not pooled with the study data, treated as controls, or included in association testing. Approximate heterozygote frequencies and available allele-frequency estimates are summarized in Supplementary Table S4.

Expression quantitative trait locus data for rs755622–MIF were reviewed using the GTEx Portal, Analysis V8, and cross-checked against the published analysis by Alban et al. [25,26]. GTEx single-tissue NES values are oriented to the alternate allele; rs755622 is G>C on GRCh38, making C the effect allele for the values reported here. For selected tissues, sample size, C-allele-oriented NES, and nominal *p* value were recorded. The testis result was excluded because the p value and m value available in the source summary could not be reconciled. GTEx v8 included 838 donors, 85.3% of whom were European American, and its donor age range did not extend beyond approximately 70 years; this demographic context was considered when interpreting relevance to the Turkish older-age cohort.

The functional association network of the MIF protein was examined using the STRING database version 12.0 (STRING Consortium). MIF was entered as the seed protein, and the analysis was restricted to *Homo sapiens* (NCBI taxonomy identifier 9606). A full STRING network was generated using evidence-based edge presentation, the default medium-confidence interaction threshold of 0.400, a maximum of 10 first-shell interactors, and no second-shell interactors. All available interaction evidence channels, including curated databases, experimentally determined associations, co-expression, text mining, genomic neighborhood, gene fusion, and gene co-occurrence, were retained. Predicted functional partners, combined STRING scores, the complete network edge list, and node-degree values were exported for analysis. STRING edges were interpreted as biologically meaningful functional or physical protein associations and not necessarily as evidence of direct molecular binding [27]. A single-protein query in STRING is expanded by default to the 10 highest-ranking functional partners, and STRING associations may represent either physical interactions or indirect functional relationships.

The variant annotation, ENCODE-informed chromatin context, tissue-specific eQTL, and protein-association-network findings were interpreted together as external biological context for the genotype-level observations. These complementary resources support biological plausibility at the locus and pathway levels but do not by themselves establish a cohort-specific effect of rs755622 on *MIF* expression, *MIF* protein concentration, promoter activity, or aging-related phenotypes.

## 5. Conclusions

The present findings indicate an exploratory genotype-specific pattern involving *MIF* rs755622 and age-group status in the sampled Turkish cohort rather than evidence of a risk or protective allele. These findings pertain to cross-sectional age-group differences and should not be interpreted as evidence of an effect on aging trajectories, survival, or longevity. GC frequency showed an ordered-group trend, and conventional, Firth, exact, and sex-stratified sensitivity analyses were broadly consistent with a genotype-distribution difference; however, no result remained significant after a single global FDR or Bonferroni correction across 26 main association tests. Sex-stratified HWE analysis showed that the younger-control disequilibrium was concentrated in men, while younger women did not deviate significantly, narrowing the diagnostic pattern without identifying its cause. Deterministic and scenario-based Monte Carlo reclassification analyses further demonstrated substantial fragility of the nominal association to control genotype classification. Therefore, the observed genotype pattern should be regarded as hypothesis-generating rather than as robust evidence of association pending independent verification of the younger-control genotypes. Functional triangulation across VEP, ENCODE-informed chromatin context, GTEx, and STRING supports a biologically plausible regulatory and immune-metabolic context, but it does not establish a cohort-specific molecular effect. The present data do not establish rs755622 as a causal functional variant and cannot exclude linkage disequilibrium or population-stratification explanations. Independent genotype verification, ancestry-matched replication, broader *MIF* promoter haplotype analysis, prospective phenotyping, and direct molecular assessment remain the appropriate next steps.

## Figures and Tables

**Table 1:** Demographic characteristics of the study population.

Group	*n*	Age Range, Years	Mean Age, Years	Women, *n* (%)	Men, *n* (%)
Younger controls	123	20–46	43.0	55 (44.7)	68 (55.3)
Overall older group	245	65–102	83.5	147 (60.0)	98 (40.0)
65–89 years	205	65–89	—	—	—
≥90 years	40	90–102	—	—	—

**Table 2:** Genotype distributions and exact Hardy–Weinberg equilibrium results.

Variable	Younger Controls (*n* = 123)	Overall Older (*n* = 245)	65–89 Years (*n* = 205)	≥90 Years (*n* = 40)
GG, *n* (%)	108 (87.8)	196 (80.0)	167 (81.4)	29 (72.5)
GC, *n* (%)	10 (8.1)	44 (18.0)	34 (16.6)	10 (25.0)
CC, *n* (%)	5 (4.1)	5 (2.0)	4 (2.0)	1 (2.5)
Exact HWE *p* value	<0.001	0.188	0.238	1.000

**Table 3:** Age-stratified genotype and allele distributions of the *MIF* rs755622 polymorphism compared with the younger control group.

Comparison	Variable	OR (95% CI)	Nominal *p*	Global FDR q
Overall older group vs. younger controls	GG	0.556 (0.298–1.037)	0.080	0.199
Overall older group vs. younger controls	GC	2.474 (1.199–5.104)	0.012	0.062
Overall older group vs. younger controls	CC	0.492 (0.140–1.732)	0.312	0.427
Overall older group vs. younger controls	C allele	1.400 (0.818–2.396)	0.244	0.373
65–89 years vs. younger controls	GG	0.610 (0.320–1.163)	0.163	0.296
65–89 years vs. younger controls	GC	2.247 (1.068–4.728)	0.030	0.111
65–89 years vs. younger controls	CC	0.470 (0.124–1.783)	0.304	0.427
65–89 years vs. younger controls	C allele	1.290 (0.738–2.253)	0.410	0.508
≥90 years vs. younger controls	GG	0.366 (0.152–0.882)	0.027	0.111
≥90 years vs. younger controls	GC	3.767 (1.436–9.882)	0.010	0.062
≥90 years vs. younger controls	CC	0.605 (0.069–5.339)	1.000	1.000
≥90 years vs. younger controls	C allele	1.994 (0.928–4.287)	0.084	0.199

Genotype-specific odds ratios compare the indicated genotype with the other two genotypes; allelic odds ratios compare C with G. Nominal two-sided Fisher tests are shown. Global FDR values were calculated across the 26 main association tests. Global Bonferroni-adjusted values are reported in Supplementary Table S2. CI, confidence interval; FDR, false discovery rate; OR, odds ratio.

**Table 4:** Crude and sex-adjusted associations between *MIF* rs755622 genetic models and older age-group status.

Genetic Model	Contrast	Crude OR (95% CI)	Nominal *p*	Sex-Adjusted OR (95% CI)	Adjusted *p*	Global FDR q
Codominant	GC vs. GG	2.424 (1.173–5.009)	0.018	2.575 (1.237–5.363)	0.011	0.062
Codominant	CC vs. GG	0.551 (0.156–1.946)	0.504	0.645 (0.180–2.320)	0.502	0.567
Dominant	GC + CC vs. GG	1.800 (0.964–3.360)	0.080	1.946 (1.033–3.666)	0.039	0.113
Recessive	CC vs. GG + GC	0.492 (0.140–1.732)	0.312	0.566 (0.158–2.024)	0.381	0.495
Overdominant	GC vs. GG + CC	2.474 (1.199–5.104)	0.012	2.618 (1.259–5.444)	0.010	0.062
Additive	Per C-allele dosage	1.400 (0.818–2.396)	0.244	1.422 (0.860–2.352)	0.170	0.296

**Table 5:** Functional annotation of the *MIF* rs755622 polymorphism.

Annotation Feature	Result
Variant identifier	rs755622
Gene	*MIF*
Reference assembly	GRCh38.p14
Genomic position	chr22:23,894,205
Nucleotide substitution	G>C
Genomic region	Non-coding 5′ promoter/regulatory region
Historical nomenclature	−173G/C
Current RefSeq nomenclature	NM_002415.2:c.−270G>C
Coding consequence	None identified
Annotation context	Non-coding promoter/regulatory-region location; functional effect unvalidated

**Table 6:** Selected GTEx v8 single-tissue eQTL results for rs755622 and *MIF* expression.

Tissue	Samples, *n*	NES per C Allele	Nominal *p* Value
Esophagus–gastroesophageal junction	330	0.482	1.2 × 10^−7^
Artery–coronary	213	0.441	1.3 × 10^−4^
Artery–aorta	387	0.418	2.7 × 10^−6^
Cells–cultured fibroblasts	483	0.408	6.1 × 10^−8^
Artery–tibial	584	0.381	8.5 × 10^−8^
Whole blood	670	0.319	1.4 × 10^−6^
Lung	515	0.263	6.7 × 10^−4^
Spleen	227	0.220	0.040

NES, normalized effect size relative to the alternate C allele in GTEx single-tissue eQTL analysis. Nominal *p* values are shown without cohort-level functional validation. Only sample size, C-allele-oriented NES, and nominal *p* values are presented. The m-value field and the testis result were excluded because the available source summary did not permit internally consistent interpretation.

**Table 7:** Principal MIF-associated proteins identified using STRING v12.0.

MIF-Associated Protein	STRING Combined Score	Node Degree	Principal Functional Context
CD74	0.999	4	MIF receptor; MHC class II antigen processing
CXCR2	0.993	4	Chemokine signaling; neutrophil recruitment
CXCR4	0.991	4	Chemokine signaling; migration; MAPK/AKT-associated responses
COPS5	0.976	4	COP9 signalosome; ubiquitin-related and cellular stress regulation
GOT2	0.929	6	Mitochondrial aminotransferase; kynurenine and amino-acid metabolism
GOT1	0.915	6	Cytosolic aspartate/glutamate metabolism
TAT	0.910	6	Tyrosine catabolism
HPD	0.909	6	Tyrosine degradation
GOT1L1	0.907	6	Putative cytosolic aminotransferase activity
IL4I1	0.902	6	Amino-acid oxidation; lysosomal antigen processing

## Data Availability

The aggregated data supporting the findings of this study are contained within the article and its Supplementary Materials.

## Supplementary Materials

The following supporting information can be downloaded at https://www.mdpi.com/article/10.3390/ijms27177818/s1.
