# Supplementary material for: MIF Promoter Variant rs755622 (−173G/C) in Younger and Older Turkish Adults: An Exploratory Cross-Sectional Genetic and in Silico Analysis
Source: Int J Mol Sci. 2026 Aug 31;27(17):7818. doi: 10.3390/ijms27177818 (PMC13566417; doi:10.3390/ijms27177818)
Supplement: Supplementary file 1 [file ijms-27-07818-s001.zip › ijms-4474489-supplementary.pdf]

# Supplementary Materials

## Table S1. Deterministic sensitivity analysis of the overdominant association under hypothetical younger-control genotype reclassification

In the younger control group, the observed allele frequencies were G = 226/246 (0.9187) and C = 20/246 (0.0813). Under Hardy–Weinberg equilibrium, the expected genotype counts were approximately GG = 103.82, GC = 18.37, and CC = 0.81, compared with observed counts of 108, 10, and 5. Each scenario below hypothetically transfers one younger-control participant from the non-GC category to the GC category while the older-group counts remain fixed at 44 GC and 201 non-GC. Scenarios were extended through eight reclassifications, approximately spanning the observed heterozygote deficit relative to the HWE expectation. The observed dataset is unchanged, and this analysis does not replace independent genotype verification.

| Hypothetical reclassifications (non-GC to GC) | Younger GC, n | Younger non-GC, n | Overdominant OR | Two-sided Fisher p |
|---|---|---|---|---|
| 0 | 10 | 113 | 2.474 | 0.012 |
| 1 | 11 | 112 | 2.229 | 0.029 |
| 2 | 12 | 111 | 2.025 | 0.045 |
| 3 | 13 | 110 | 1.852 | 0.068 |
| 4 | 14 | 109 | 1.704 | 0.129 |
| 5 | 15 | 108 | 1.576 | 0.177 |
| 6 | 16 | 107 | 1.464 | 0.295 |
| 7 | 17 | 106 | 1.365 | 0.373 |
| 8 | 18 | 105 | 1.277 | 0.463 |

OR, odds ratio. Fisher *p* values are two-sided and are shown to three decimal places.

## Table S2. Global multiplicity correction across 26 main association tests

The global family comprised 12 age-group genotype-specific or allelic comparisons, six sex-adjusted inheritance-model comparisons, six sex-stratified genotype comparisons, one ordered-group trend test, and one genotype-by-sex interaction test. For age-group, sex-stratified, trend, and interaction analyses, the input value was the nominal p value; for inheritance models, it was the sex-adjusted p value. The six Firth analyses in Table S3 re-estimated the same inheritance-model hypotheses and were not counted as additional tests. The analyses in Table S6 were treated as exploratory sensitivity re-estimations outside the defined 26-test family, and Table S7 reports diagnostic HWE tests rather than association hypotheses. Adjusted values were calculated from unrounded p values, whereas displayed input p values are rounded. No test remained significant after global false-discovery-rate or Bonferroni correction.

| Analysis family | Comparison or contrast | Input p value | Global FDR q | Global Bonferroni-adjusted p |
|---|---|---|---|---|
| Age-group | Overall older vs. younger: GG | 0.080 | 0.199 | 1.000 |
| Age-group | Overall older vs. younger: GC | 0.012 | 0.062 | 0.312 |
| Age-group | Overall older vs. younger: CC | 0.312 | 0.427 | 1.000 |
| Age-group | Overall older vs. younger: C allele | 0.244 | 0.373 | 1.000 |
| Age-group | 65–89 vs. younger: GG | 0.163 | 0.296 | 1.000 |
| Age-group | 65–89 vs. younger: GC | 0.030 | 0.111 | 0.780 |
| Age-group | 65–89 vs. younger: CC | 0.304 | 0.427 | 1.000 |
| Age-group | 65–89 vs. younger: C allele | 0.410 | 0.508 | 1.000 |
| Age-group | ≥90 vs. younger: GG | 0.027 | 0.111 | 0.702 |
| Age-group | ≥90 vs. younger: GC | 0.010 | 0.062 | 0.260 |
| Age-group | ≥90 vs. younger: CC | 1.000 | 1.000 | 1.000 |
| Age-group | ≥90 vs. younger: C allele | 0.084 | 0.199 | 1.000 |
| Sex-adjusted inheritance model | Codominant GC vs. GG | 0.011 | 0.062 | 0.286 |
| Sex-adjusted inheritance model | Codominant CC vs. GG | 0.502 | 0.567 | 1.000 |
| Sex-adjusted inheritance model | GC + CC vs. GG | 0.039 | 0.113 | 1.000 |
| Sex-adjusted inheritance model | CC vs. GG + GC | 0.381 | 0.495 | 1.000 |

| | | | | |
|---|---|---|---|---|
| Sex-adjusted inheritance model | GC vs. GG + CC | 0.010 | 0.062 | 0.260 |
| Sex-adjusted inheritance model | Per C-allele dosage | 0.170 | 0.296 | 1.000 |
| Sex-stratified | Men GG | 0.171 | 0.296 | 1.000 |
| Sex-stratified | Men GC | 0.034 | 0.111 | 0.884 |
| Sex-stratified | Men CC | 0.446 | 0.527 | 1.000 |
| Sex-stratified | Women GG | 0.188 | 0.305 | 1.000 |
| Sex-stratified | Women GC | 0.163 | 0.296 | 1.000 |
| Sex-stratified | Women CC | 1.000 | 1.000 | 1.000 |
| Ordered trend | GC across 20–46, 65–89, ≥90 | 0.004 | 0.062 | 0.108 |
| Interaction | Genotype × sex | 0.815 | 0.883 | 1.000 |

FDR, false discovery rate. The Bonferroni value for the ordered trend is 0.108 because adjustment used the unrounded two-sided *p* value (0.0041629), not the rounded display value of 0.004.

## Table S3. Sex-adjusted Firth penalized logistic-regression sensitivity analysis

Older-versus-younger age-group status was the outcome, genotype contrast was the predictor, and sex was included as a covariate. Firth estimates used Jeffreys-prior penalized likelihood to reduce sparse-data bias. The *p* values are nominal sensitivity-analysis values and were not added as separate hypotheses to the 26-test multiplicity family. These estimates do not correct genotype misclassification or control-group Hardy–Weinberg disequilibrium. CI, confidence interval; OR, odds ratio.

| Model | Contrast | Firth OR (95% Wald CI) | Two-sided Wald p |
|---|---|---|---|
| Codominant | GC vs. GG | 2.480 (1.201–5.121) | 0.014 |
| Codominant | CC vs. GG | 0.647 (0.180–2.324) | 0.504 |
| Dominant | GC + CC vs. GG | 1.902 (1.014–3.571) | 0.045 |
| Recessive | CC vs. GG + GC | 0.568 (0.159–2.030) | 0.384 |
| Overdominant | GC vs. GG + CC | 2.521 (1.222–5.200) | 0.012 |
| Additive | Per C-allele dosage | 1.396 (0.847–2.302) | 0.191 |

## Table S4. Descriptive external rs755622 genotype and allele frequency benchmarks

External values are descriptive benchmarks rather than formal controls because recruitment, phenotype, ancestry, and genotyping or sequencing methods differ. The 1000 Genomes EUR/EAS/SAS entry reports the approximate GC heterozygote-frequency range across those superpopulations rather than a pooled estimate; TSI (Toscani in Italy) is shown separately as a population-specific European benchmark. A dash indicates that the corresponding genotype or allele-frequency value was not available in the summarized benchmark. These comparisons contextualize the unusually low younger-control GC and C-allele frequencies but do not resolve the Hardy–Weinberg disequilibrium. Reference numbers correspond to the main manuscript.

| Source | Population | Sample size, n | GG, % | GC, % | CC, % | C allele, % |
|---|---|---|---|---|---|---|
| Present study | Younger controls | 123 | 87.8 | 8.1 | 4.1 | 8.1 |
| Present study | Overall older | 245 | 80.0 | 18.0 | 2.0 | 11.0 |
| 1000 Genomes Phase 3 [35] | EUR/EAS/SAS superpopulations | 503/504/489 | — | ≈31–33 | — | — |
| 1000 Genomes Phase 3 [35] | TSI (Toscani in Italy) | 107 | — | — | — | 18.2 |
| gnomAD [36] | Non-Finnish European | — | 65.2 | 31.7 | 3.1 | 19.0 |
| Akcali et al. [33] | Turkish controls | 120 | 55.0 | 40.8 | 4.2 | 24.6 |
| Pehlivan et al. [34] | Turkish controls | 67 | 65.7 | 31.3 | 3.0 | 18.7 |

EUR, European; EAS, East Asian; SAS, South Asian; TSI, Toscani in Italy. Approximate symbols and dashes are retained to avoid implying precision or data fields not available in the summarized external benchmark. For TSI, the C-allele frequency (18.2%) was calculated from the 1000 Genomes Project Phase 3 allele-frequency output (39 C alleles among 214 total alleles).

## Table S5. Exploratory probabilistic genotype-misclassification bias analysis (scenario-based Monte Carlo sensitivity analysis)

This scenario-based Monte Carlo analysis summarizes the same younger-control reclassification range examined deterministically in Table S1. Because independent validation data were not available to specify empirically calibrated genotype-call sensitivity or specificity distributions, K, the number of potentially hidden GC calls among the 113 observed younger-control non-GC genotypes, was assigned a discrete uniform distribution over the integers 0–8. The upper bound approximately corresponds to the observed heterozygote deficit relative to the HWE expectation (18.37 expected GC versus 10 observed). In each of 100,000 iterations (seed = 20260807), older-group counts were fixed at 44 GC and 201 non-GC and younger-control counts were set to 10 + K GC and 113 − K non-GC. The cross-product OR and two-sided Fisher exact p value were calculated in each iteration. The percentile range below reflects the distribution generated by this specified hypothetical scenario model and is not a confidence interval or an empirically calibrated genotype-error estimate [37].

| Parameter or result | Value |
|---|---|
| Scenario parameter | K = potentially hidden GC calls among observed younger-control non-GC genotypes |
| Distribution for K | Discrete Uniform {0, 1, 2, ..., 8} |
| Monte Carlo iterations | 100,000 |
| Random seed | 20260807 |
| Older-group counts held fixed | 44 GC; 201 non-GC |
| Median sampled K | 4 |
| Median simulated OR | 1.704 |
| Mean simulated OR | 1.774 |
| 2.5th–97.5th percentile range of simulated OR | 1.277–2.474 |
| Median two-sided Fisher p | 0.129 |
| Iterations with Fisher p < 0.05 | 33.3% |

Under the equal-probability 0–8 reclassification model, the nominal association remained $p < 0.05$ in approximately one-third of simulations. The simulation therefore quantifies sensitivity to explicit genotype-reclassification scenarios rather than estimating a true misclassification rate or a corrected effect OR, odds ratio.

## Table S6. Additional exploratory model-free, exact-trend, and sex-stratified sensitivity analyses

These exploratory analyses used the same genotype counts as the main analyses to evaluate robustness to model specification and asymptotic assumptions. They were not included in the defined 26-test multiplicity family. All p values in this table are nominal and descriptive and should not be interpreted as additional multiplicity-adjusted evidence or as independent replication of the main findings. The analyses re-estimate patterns within the same underlying genotype calls and therefore do not determine the source of the younger-control HWE disequilibrium.

| Analysis | Result or contrast | Two-sided p | Interpretation |
|---|---|---|---|
| Fisher–Freeman–Halton exact test | Overall older vs. younger 2 × 3 genotype table (GG/GC/CC) | 0.022 | Model-free nominal difference in overall genotype distribution. |
| Exact conditional permutation trend | GC across 20–46, 65–89, and ≥90 years; observed score statistic T = 54 | 0.0047 | Closely matched the asymptotic Cochran–Armitage result; same ordered-trend hypothesis. |
| Cochran–Mantel–Haenszel analysis | GC vs. GG + CC, stratified by sex; common OR = 2.606 (95% CI: 1.254–5.413) | 0.0083 | Directionally concordant stratified estimate of the overdominant contrast. |
| Breslow–Day homogeneity test | Homogeneity of male and female overdominant odds ratios | 0.815 | No evidence that the overdominant association differed between sex strata. |

CI, confidence interval; OR, odds ratio. The Fisher–Freeman–Halton two-sided exact *p* value was obtained by enumerating all 2 × 3 tables with fixed margins and summing probabilities no greater than that of the observed table. The exact trend *p* value was obtained from the conditional distribution of GC carriers across the three mutually exclusive age categories. The Cochran–Mantel–Haenszel and Breslow–Day results use the male and female genotype counts reported in Table 5 of the main manuscript.

## Table S7. Sex-stratified exact Hardy–Weinberg equilibrium analysis

Exact HWE was evaluated within male and female strata for the younger controls and the overall older group using the sex-specific genotype counts reported in Table 5 of the main manuscript. These tests are diagnostic evaluations of genotype-distribution quality and were not included in the 26-test association multiplicity family.

| Stratum | Observed GG / GC / CC | C-allele frequency | Exact HWE p |
|---|---|---|---|
| Younger men (n = 68) | 58 / 6 / 4 | 0.1029 | 0.00094 |
| Younger women (n = 55) | 50 / 4 / 1 | 0.0545 | 0.134 |
| Overall older men (n = 98) | 74 / 21 / 3 | 0.1378 | 0.382 |
| Overall older women (n = 147) | 122 / 23 / 2 | 0.0918 | 0.342 |

HWE, Hardy–Weinberg equilibrium. Exact p values were calculated using the Wigginton exact-test algorithm. The younger-control disequilibrium was concentrated in the male stratum, whereas the younger female and both older sex strata showed no significant deviation. This diagnostic pattern does not by itself identify whether the underlying source reflects genotype-classification uncertainty, sampling variation, batch structure, or population substructure.

## Table S8. Exploratory sex-stratified genotype distributions and association estimates for MIF rs755622

| Sex | Genotype | Older, n (%) | Younger, n (%) | OR (95% CI) | Nominal p | Global FDR q | Global Bonferroni-adjusted p |
|---|---|---|---|---|---|---|---|
| Men | GG | 74 (75.5) | 58 (85.3) | 0.532 (0.236–1.200) | 0.171 | 0.296 | 1.000 |
| Men | GC | 21 (21.4) | 6 (8.8) | 2.818 (1.072–7.412) | 0.034 | 0.111 | 0.884 |
| Men | CC | 3 (3.1) | 4 (5.9) | 0.505 (0.109–2.334) | 0.446 | 0.527 | 1.000 |
| Women | GG | 122 (83.0) | 50 (90.9) | 0.488 (0.177–1.347) | 0.188 | 0.305 | 1.000 |
| Women | GC | 23 (15.6) | 4 (7.3) | 2.365 (0.779–7.181) | 0.163 | 0.296 | 1.000 |
| Women | CC | 2 (1.4) | 1 (1.8) | 0.745 (0.066–8.383) | 1.000 | 1.000 | 1.000 |

Genotype-specific odds ratios compare the indicated genotype with the other two genotypes within each sex stratum. Nominal two-sided Fisher exact p values are shown. Global FDR q values and Bonferroni-adjusted p values were calculated within the prespecified family of 26 main association tests. No sex-stratified comparison remained statistically significant after global multiplicity correction. Because several sex-by-genotype cells contained few participants, these exploratory subgroup estimates should be interpreted cautiously. The formal genotype-by-sex interaction under the overdominant model was also non-significant (p = 0.815; global FDR q = 0.883; Bonferroni-adjusted p = 1.000). OR, odds ratio; CI, confidence interval; FDR, false discovery rate.
